# Institutional multiplexity: social networks and community-based natural resource management

**DOI:** 10.1007/s11625-018-0549-2

**Published:** 2018-03-15

**Authors:** Michael Schnegg

**Affiliations:** Institute of Social and Cultural Anthropology, Universität Hamburg, 20146 Hamburg, Germany

**Keywords:** Social networks, Social network analysis, Natural resource governance, CBNRM, Water, Africa

## Abstract

Natural resource management has changed profoundly in recent decades emphasizing new legislation that transfers responsibilities to local user groups. In this article, I follow changing water policies to Namibia and show that the enactment of policy in local institutions deviates from community-based natural resource management (CBNRM) blueprints and design. To understand why, I examine the theoretical premises of CBNRM. CBNRM is informed by rational choice theory which isolates economic transactions (e.g., sharing water) and assumes that people design institutions for a specific good. However, in the communities I study ethnographically, people depend on sharing multiple resources. To better understand how the degree of sharing and institutional overlaps matter, I explore empirically the concept of institutional multiplexity. Institutional multiplexity describes the number of transactions between two households in a social network. The results reveal that almost all social networks are institutionally multiplex. Institutional multiplexity implies that people cannot separate the sharing of water from sharing in other domains. Institutional multiplexity hinders the implementation of design principles such as fixing boundaries, sharing costs proportional to use, and formal sanctioning. However, it also opens other means for governing nature through social control.

## Introduction

In recent decades, the ways states regulate natural resources has profoundly changed. It has become a central theme of many national policies to transfer the responsibility for managing environmental resources to local communities though formal organizations. The community-based natural resource management (CBNRM) approach that is often applied in such cases is underpinned by two theoretical frameworks: first, common-pool resource (CPR) theory, which has shown that local communities are able to manage resources successfully over long periods of time (Acheson 1989; Berkes et al. 1989; Bromley et al. 1992; McCabe 1990; Ostrom 1990; Wade 1994). CPR theory promises a third solution to Hardin’s (1968) “tragedy of the commons”, in addition to privatization or control by the state. Second, shifting political debates concerning economic development in the 1980s and 1990s began linking local participation, economic development, and ecological sustainability. In short, the argument runs that people’s livelihoods improve once they are empowered and able to capture benefits previously beyond their control. Once people profit economically, they have more incentives to protect their resources for sustainable use (Agrawal 2001; Dressler et al. 2010; Ribot et al. 2006).

However, as the number of community-based projects has increased, so has skepticism about their potential for success (Blaikie 2006; Büscher et al. 2014; Dressler et al. 2010). The criticism addresses both the theoretical assumptions and the negative practical consequences of the CBNRM model (Saunders 2014). For example, several scholars have argued that the underlying “model of man” as a rational and well-informed utility maximizer is not suitable to explain decision-making outside capitalistic economies. Outside market exchanges, people do not maximize returns but instead aim to reduce risks and vulnerabilities through sharing and exchange (Cashdan 1990; Cleaver 2012; Cleaver and de Koning 2015; Hall et al. 2014; Saunders 2014). Others have pointed out that CBNRM narrows the relationship with nature down to an economizing giving and taking, neglecting and eventually destroying other moral, emotional, and spiritual values people attach to their environment; those include the value landscapes have places where historic events took place, sprits live, and people feel they belong. This neglect causes severe limitations for understanding how people interact with their environment, and can have far reaching consequences when practical applications are attempted that ignore these other values (Büscher 2011; Büscher et al. 2014; Pröpper 2015; Schnegg et al. 2014; Sullivan 2009, 2014). Again others have shown that communities are more heterogeneous than the CPR theory assumes and that the power relationships within communities largely structure the success of CBNRM policies. More often than not, the withdrawal of the state opens social spaces for local elites to capture large shares of the benefits distributed in community-based management schemes, or, alternatively, to avoid their costs (Agrawal and Gupta 2005; Dasgupta and Beard 2007; Platteau 2004; Platteau and Abraham 2002; Ribot et al. 2006; Schnegg 2016b).

On an abstract level then, these criticisms relate the problems encountered with CBNRM to (1) characteristics of people (e.g., appropriate frameworks for decision-making, cultural norms and values, bargaining power) or to (2) the context in which the CBNRM model is applied (e.g., heterogeneity inequality, poverty). However, recent ethnographic evidence hints a third type of cause for failure and success: social networks and the ways they embed the economy and other social practices when CBNRM is applied (Cleaver 2002, 2012; de la Torre-Castro 2006; Mosse 2003; Schnegg and Linke 2015).

As stated, CBNRM is partly based on theoretical assumptions from rational choice theory (Cleaver 2012; Saunders 2014). Rational choice theory typically views an actor’s behavior over time as a sequence of separable choices which are made in insolation from one another. Facing one decision, such as how much to pay for water, or whether to pay at all, the actor weighs the pragmatic outcomes implied by various options and chooses accordingly (Bardhan and Ray 2006; Coleman 1990; Ostrom 1990). Ostrom (1990: 37) was critical of some aspects of rational choice theory and proposed a “very broad conception of rational action” in her seminal work. This implies that norms play a key role in explaining how individuals weigh expectations and potential costs (Ostrom 1990: 193; Vanberg 2002). At the same time, decisions focus on specific recourses (e.g., sharing water or land) which are rarely embedded in other social fields.

In recent years, these basic assumptions of rational choice theory were specified and advanced. On the one hand, the emerging system perspective in institutional analysis takes the coupling between social and biological systems explicitly into account. The looming framework first demonstrated and now acknowledges feedbacks, uncertainties, and nestedness in coupled social–ecological systems (Cox et al. 2010; Janssen et al. 2007; Scoones et al. 2007). On the other hand, Frances Cleaver and others have successfully established “critical institutionalism”, which confronts common-property resource theory with its major sociological and anthropological flaws. Cleaver and others have noted in particular the lack of an appreciation of actors’ social and cultural embeddedness (Cleaver 2002, 2012; Cleaver and de Koning 2015; de la Torre-Castro 2006; Hall et al. 2014; Mosse 2003; Schnegg and Linke 2015).^1^ To pinpoint the embeddedness of institutional development, Cleaver proposed the concept of “institutional bricolage”, which refers to a process by which people consciously and unconsciously draw on existing social and cultural arrangements to shape institutions. While both “critical institutionalism” and Cleaver’s concept of “institutional bricolage” are intellectually highly stimulating, they do not adequately offer conceptual and methodological guides for exploring embeddedness in detail in specific case studies.

To fill this gap, I link institutional analysis with social network theory and introduce the concept of institutional multiplexity. Institutional multiplexity describes the degree to which resource-based transactions, such as sharing water, are embedded in other types of network ties (e.g., sharing, food, labor, and ancestries). Since distinct sharing ties imply distinct rules, institutional multiplexity captures the number of institutions that structure the interaction between two people. The more institutionally multiplex a social tie is, the more difficult it is to isolate a single transaction and to interact as if one only shares water, land, or any other single good.^2^ As I will show, institutional multiplexity is typically high when people live in small face-to-face communities and their livelihoods depend on sharing multiple resources.

In this article, institutional multiplexity serves as a theoretical guide for exploring the decentralization of water management in rural Namibia. In presenting this view, I explore the validity of the “rational man” assumption for CBNRM research against the backdrop of multiple resource dependencies, and discuss the consequences that may follow if assumptions are not valid. In northwestern Namibia, access to water was for a long time governed by the South West Africa administration under the jurisdiction of the colonial South African state. After Independence in 1990, the Namibian state initiated major political reforms to overcome the structural inequalities of the past. Those included new legislation to govern the maintenance and distribution of the rural water supply (Falk et al. 2009; Namibia 2004; Schnegg 2016b). A shift towards self-governance was administered by the government and NGOs who developed blueprints (e.g., pre-drafted “management plans” and “constitutions” to which only a small amount of information has to be added) for how to govern water. Guided by these agencies, pastoral communities have had to develop new institutional regimes for sharing water and the costs this implies. Today, community-based water management is widely practiced in northwestern Namibia, and yet the institutions differ significantly from the governmental blueprints and CBNRM design. The aim of this article is to deploy the notion of institutional multiplexity and explain why. Before proceeding, some notes on the relationship between the economy and society are in order.

## Embeddedness of economic action

The role of economy in society has occupied anthropology for a long time (Dalton 1969; Polanyi 1944; Wilk and Cliggett 2007). Already a century ago, Malinowski surmised that economic action, such as plowing a field or sharing food, is embedded in multiple social institutions, including kinship and religion (Malinowski 1922: 60). He argued that one can hardly understand why a man gives the best share of his harvest to someone else without knowing how the two individuals are related and what this kinship relationship implies in the first man’s cultural universe (Malinowski 1922). Embeddedness and institutional overlaps became the theoretical cornerstone of the holistic paradigm in anthropology; participant observations are the practical tool to explore this.

Malinowski’s analysis was confirmed by other anthropologists of the time (Dalton 1969; Herskovits 1940). However, it took until the seminal works of Karl Polanyi to translate this perspective into a major theoretical point (Polanyi 1944). Polanyi argued that the capitalist market changed the relationship between the economic and the social significantly. Before the expansion of markets, most societies relied on reciprocity and shared distributions as primary means for transferring goods. With these institutions, the economy is per se embedded in social ties. Hence, drawing on Malinowski’s argumentation, approaches to economic behaviors as isolated actions fail to provide an adequate account of exchange. With the rising importance of markets, however, the picture began to change. Goods are now transferred between members of a society who have no personal relationships, and “price” has replaced social ties as a means to enable exchange. As a consequence, the economy became increasingly dis-embedded from social relationships (Polanyi 1944). Today, many economic sociologists would go one step further and show that even capitalistic markets do not operate outside the social field. Social networks are often a precondition for successful market exchanges because they reduce transaction costs (Granovetter 1985, 2005; Powell et al. 2005).

To summarize, anthropologists agree that the economy and society are intertwined. This is unquestionable for subsistence economies, where economic goods such as water, forests, land, and game are produced and distributed among members of a community. However, for a long time those insights did not spill over into mainstream economic theory and political science (Bardhan and Ray 2006).^3^ Therefore, it is no surprise that the CBNRM model, largely informed by economic theory, has led to institutional blueprints that conceptualize resource management as isolated tasks. To evaluate these blueprints and the assumptions on which they have been based, and to assess the consequences of assuming an independent and isolated economy in which individuals act as rational decision-makers, below I introduce social networks and the notion of institutional multiplexity.

## Social networks and institutional multiplexity

A social network is defined as a set of nodes and ties with the additional property that the emerging social structure can help to explain the behavior of the actors involved (Radcliffe-Brown 1952). By definition, the social network paradigm shifts the focus from the individual and its properties (also referred to as attributes) to the relations among them. Over recent decades, social network analysis has become a major interdisciplinary research field and has shown convincingly that it can help to explain a large range of social phenomena (Freeman 2004; Schnegg 2006; Schweizer and White 1998).

Given its overwhelming success, it is astonishing how little the social network approach has been used to study institutional development and natural resource management until very recently (Bodin 2017; Henry and Vollan 2014). Few examples are an exception to that rule. The work of Fliervoet and colleagues, for example, analyzes the floodplain management in the Dutch Rhine delta and the consequences of abolishing central actors in the organizational networks that govern the process (Fliervoet et al. 2016). In a similar vein, Lyle and Smith (2014) examine which network factors contribute to collective action in an Andean community and find that cooperative households have better reputations for various qualities and larger support networks (Lyle and Smith 2014). Moreover, Bodin and Crona (2008) report that high levels of social capital (measured in network terms) do not always predict sustainable resource use (Bodin and Crona 2008).

Most existing studies that link social networks and resource management focus on the internal structure of user communities (e.g., measured through connectivity and density) and the ways those network properties enable or hinder common-property management (Bodin and Crona 2008; Janssen et al. 2006). However, to explore the integration of resource economies and other social fields, dyadic relationships between the members of a community are more salient than overall network properties. As I will show, the network concept of multiplexity can serve as a guide to capture the most important property of dyadic ties and overlapping social fields (Haythornthwaite 2001; McPherson et al. 2001; Schweizer et al. 1996; Verbrugge 1979).

The idea of multiplexity (and its opposite, uniplexity) goes back to Simmel’s image of social circles (*soziale Kreise*) and the sociological theory of roles (Dahrendorf 2006; Nadel 1957; Simmel 1992 [1908]). Comparing rural and urban livelihoods, these scholars argued that these lifeways differ significantly by the extent to which social roles and relationships overlap. While they overlap significantly in some societies, with urbanization and economic differentiation overlaps tend to decline.

**Fig. 1:**
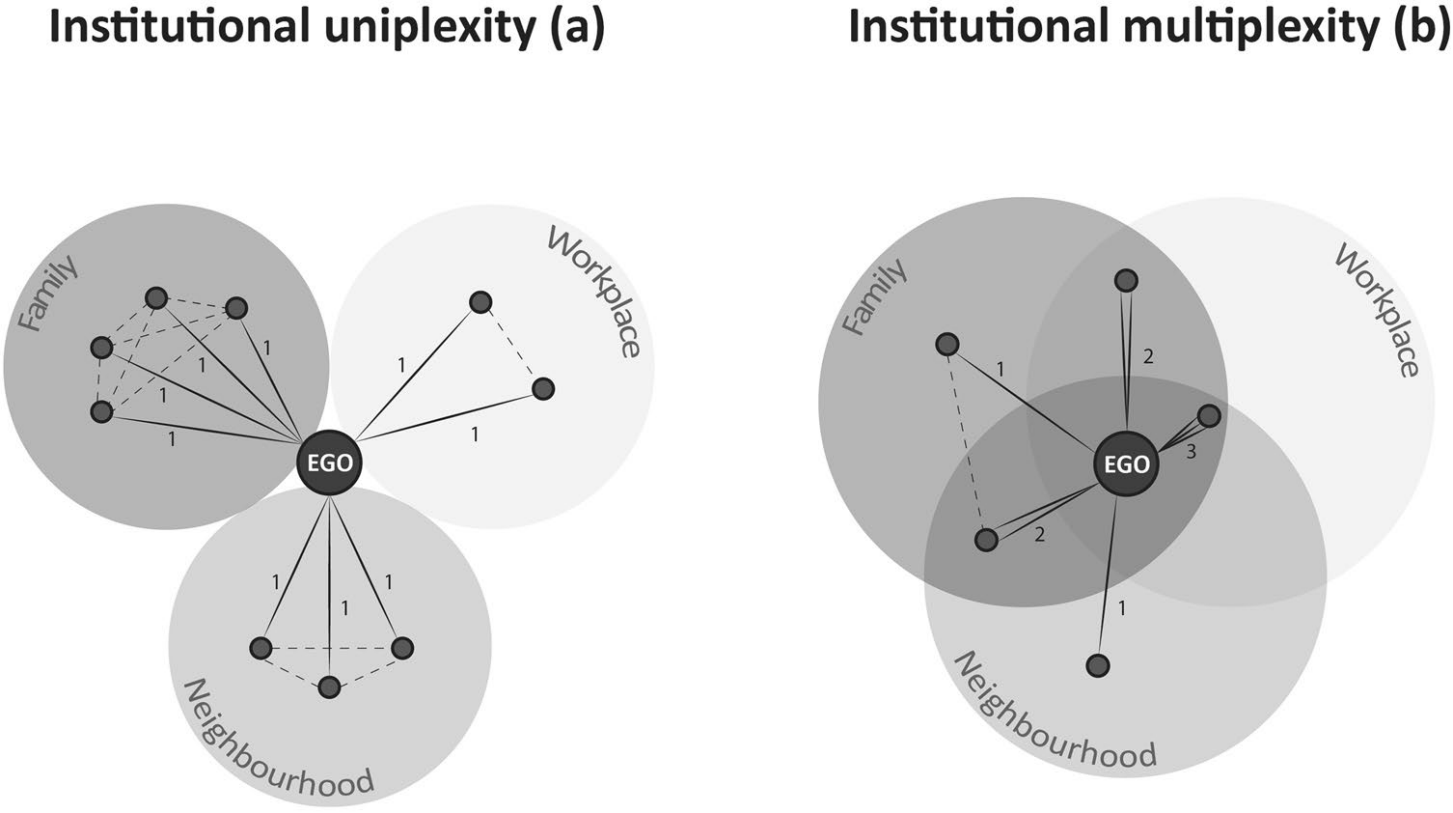
Uniplex (**a**) and multiplex (**b**) social network in comparison

The anthropologist Max Gluckman did not cite Simmel’s work when he coined the term *multistrain*, or *multiplex* to refer to the social processes he observed when studying conflict resolution in rural Rhodesia. Gluckman argued that traditional judges and the people they interact with are not only related through their immediate roles. They are, at the same time, neighbors, kin, and co-workers. This normative multiplicity restricts the agency of the judge in some ways. At the same time, it offers alternative opportunities to investigate delinquencies and to settle conflicts (Gluckman 1955). Bruce Kapferer, a student of Gluckman, further formalized the idea and defined *multiplexity* as the number of social relationships that exists between two people in a complete network (Kapferer 1969). The social configurations in Fig. 1 use the contrast between urban and rural livelihoods to exemplify what institutional uniplexity and multiplexity imply.

In Fig. 1a, the uniplex network of an idealized urban folk is shown. Here, EGO, the person in focus, interacts in three different social fields: workplace, family, and neighborhood. In each of these fields, EGO interacts with different people and the social network that results is completely uniplex (indicated by the multiplexity value = 1 on the ties). All relationships contain different transactions, but in only one domain. In contrast, the network in Fig. 1b pictures EGO as a prototypical rural dweller, and his or her relationships are multiplex. Here, the same person, EGO, interacts with the same people in different contexts. The social circles overlap and the same person is a co-worker, a neighbor, and family (again indicated by the multiplexity values on the ties). Each of these roles comes with certain expectations and is governed by institutional norms and rules. For example, solidarity, trust, and expectations of reciprocation differ between a brother and a boss. If we interact with different people in different roles (institutional uniplexity), it is comparably easy to keep those norms and expectations (aka institutions) apart. By contrast, if these roles start to overlap, it becomes impossible to single out one relationship alone (institutional multiplexity). If the boss is also a brother to me, it is impossible to interact only as family members or only as boss and employee, either at home or at work. The concept of institutional mutiplexities captures and measures the overlap of different institutional regimes that emerges if two people interact in more than one way.

Put formally and borrowing the notation from network theory, *G*(*V*,[*Ri*]) is a multirelational network with vertex set *V* and relations [*Ri*]. If *v* and *w* are two vertices of G then the multiplexity of the relationships is defined as the sum all relations connecting *v* and *w*. The index of institutional multiplexity varies between zero and the number of relations [*Ri*].

In the case of resource management, the sharing of resources such as water or land often constitutes only one tie among many between two people. Typically, those connections overlap with other social domains. Institutional multiplexity becomes a way to describe and access the social embeddedness of the economy systematically. As I show below, institutional multiplexity is a major reason why CBNRM is not practiced in rural communities in the ways it had been designed by outsiders. Some background on the ethnography that provides the basis for my research is in order, however, before proceeding.

## The ethnographic scene

Pastoralism is the dominant subsistence strategy in the Kunene region of northwestern Namibia, where dependency on multiple natural resources is high. Pastoral livelihoods are constrained by low and unpredictable precipitation, which mostly occurs in summer, between November and April. Under these environmental constraints, vast lands are needed to keep livestock (Burke 2004; Schnegg and Bollig 2016).

In the pastoral communities throughout Kunene, cattle, goats, and sheep are the heaviest water consumers. Until some 50 years ago, most pastoralists obtained water through natural springs, surface water, and hand-dug wells. This picture changed in the middle of the twentieth century when the colonial state started drilling hundreds of boreholes on communal lands (Bollig 2013). Today, water is largely provided through boreholes. During colonial rule, the management of these boreholes was largely accomplished by the administration of South West Africa under the jurisdiction of the colonial South African state. As long as the state covered the costs for establishing, running, and maintaining the infrastructure little, local coordination was required. Water was basically free (Schnegg 2016b; Schnegg and Bollig 2016; Schnegg and Linke 2015).

This situation changed in the 1990s when a new water policy entered the stage. Inspired by the idea of CBNRM, the independent Namibian state turned the responsibility and partly also the ownership of central natural resources over to local user associations (Bollig and Menestrey Schwieger 2014; Falk et al. 2009; Schnegg and Bollig 2016). This paradigm shift was not restricted to water but applied to wildlife, which was to become managed in communal conservancies, as well (Jones and Weaver 2009; Naidoo et al. 2016; Nuulimba and Taylor 2015; Sullivan 2003). During this organizational and institutional change, pastoral communities had to develop “new” institutions for how to share the costs and the benefits accruing from water sources under their management. The negotiation of these regimes did not take place in an ideological vacuum but was largely shaped by the ideas of CBNRM and structured by NGO and state representative associations (Blaikie 2006; Silva and Mosimane 2012; Vette et al. 2012).

In particular, the community-based water policy and the blueprints that circulated to implement them includes three assumptions: (1) the notion of a fixed and bounded user group, (2) the idea that those who use more should contribute more, and (3) the notion that formal sanctions are necessary to ensure the working of institutional regimes.

## Methods and data

**Fig. 2:**
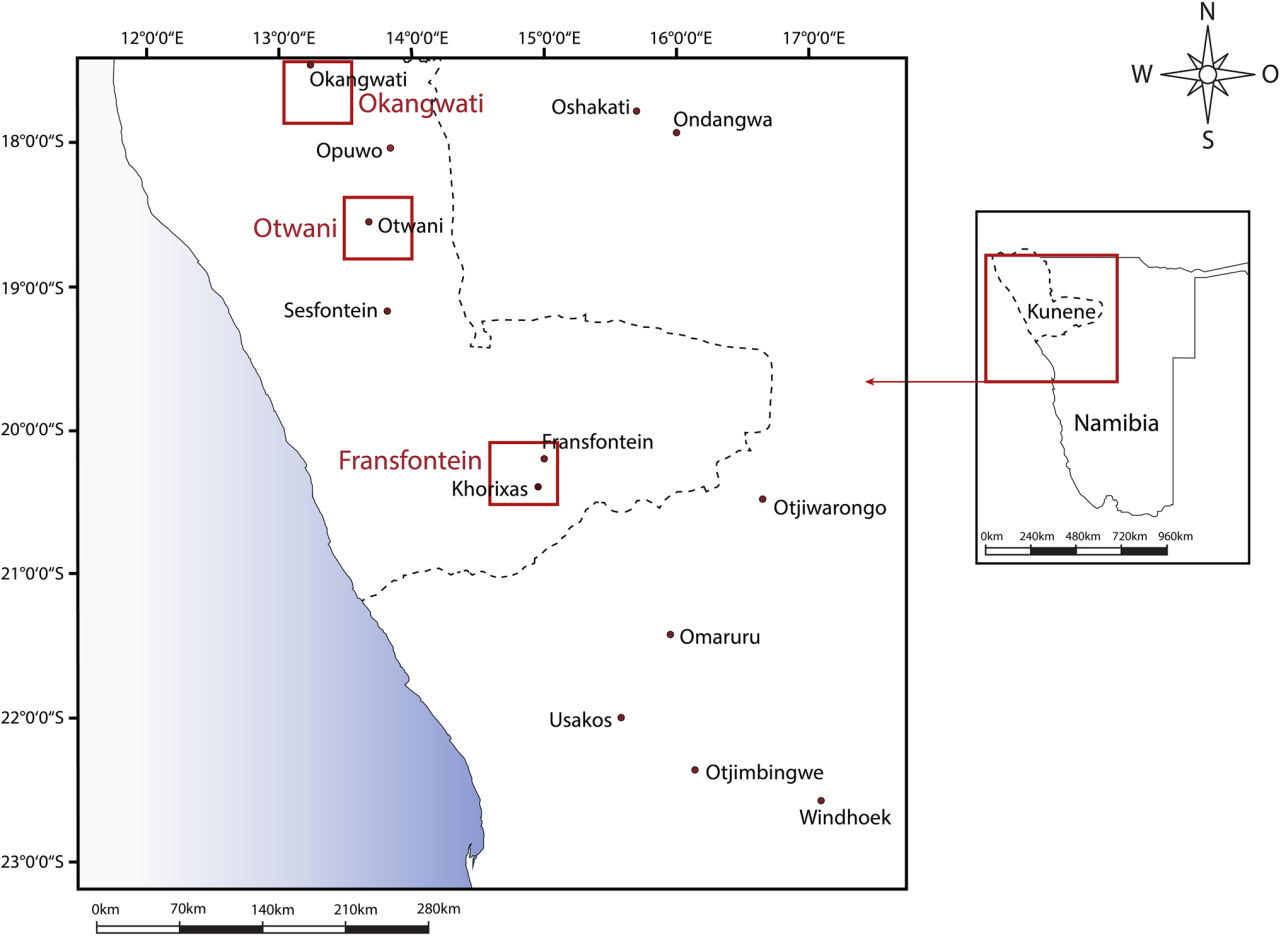
Research area in northwestern Kunene, Namibia

The data analyzed here were collected in northwestern Namibia by a team of eight anthropologists (Bollig, Dimba-Kiaka, Gradt, Kelbert, Linke, Menestrey-Schwieger, Olwage, and Schnegg) from 2010 onwards as part of the German Research Council (DFG) funded research project LINGS (Local Institutions in Globalized Societies, http://www.lings-net.de). The two principle investigators, Schnegg and Bollig, have conducted ethnographic fieldwork in the region since 1994 (Bollig) and 2003 (Schnegg), respectively, and are responsible for the overall design and the comparative analysis of the data. In the first phase of the current fieldwork, three anthropologists (Gradt, Linke, Menestrey-Schwieger) stayed for roughly 1 year in the southern (Fransfontein), central (Otwani), and northern (Okangwati) parts of the research area to gain an in-depth understanding of the process of negotiating and crafting new institutions in daily routines (Linke 2017; Menestrey Schwieger 2015, 2017). Again, three anthropologists continued their work in 2014 in the same communities (Dimba-Kiaka, Olwage, Menestrey-Schwieger). Since the communities are small (between eight and 17 households), we were able to investigate seven communities in detail (see Fig. 2).

To explore the social life of water, data were gathered in a mixed-method research design through participant observation, qualitative interviews, and surveys. To identify salient properties of the social structure and institutional multiplexities, we conducted a social network survey with all households of the seven communities (Schweizer et al. 1996; Wasserman and Faust 1994). To allow for comparability, we selected a core of eight relationships that were the subject of elicitation in all communities (see Table 1).

**Table 1:** Social network relationships elicited in seven communities

	Question
1	If anyone in your household needs to organize a donkey cart for the following day, whom do you ask for it?
2	If you (your house) need sugar or cooking oil, whom do you usually ask to give you some?
3	If you (your house) slaughter a goat, to whom do you send some meat?
4	Who is herding your cattle if you and your sons are absent or sick?
5	Imagine you are sick. To whom do you commit money to bring you some medicine from Fransfontein/ Otwani/ Opuwo?
6	With whom do you usually visit to have a chat?
7	If you are in urgent need of cash for paying the water fees, whom do you ask to lend you some money?
8	If you notice that the water point (all the water infrastructure) has been damaged, whom do you contact first?

All interviews (*N* = 80) were preferably conducted with the household head (male or female) or, in cases where the head was part of a couple, with his or her spouse, or with both members together (Schnegg and Linke 2015). We excluded households that subsist on part-time farming and whose members live and work in the urban centers while a shepherd stays on the farms. Although these households are often related to the households of other farmers, the day-to-day social interactions of their members cannot be compared with those of household members living in one place. For the network survey, all households we approached completed the interview.

The eight relationships elicited in the network survey corresponded to dimensions of support addressed in many international social surveys (e.g., ISSP) and were translated for the regional cultural context. They were intended to capture details of institutional, economic, and emotional sharing relationships (Freeman and Ruan 1997; Schweizer et al. 1996; Wellman and Wortley 1990). The people interviewed were free to name members of the community and outsiders (from other villages). The design thus resembled a personal network approach in which the social embedding of individuals takes center stage. For the multiplexity analysis, we utilized all dyadic relationships, both within the communities and with outsiders.

To gain a better understating of the distribution of some phenomenon beyond the seven communities, we scaled up our research. For geographical areas of approximately 250 km^2^ surrounding Fransfontein, Otwani, and Okangwati, we sampled 60 communities concentrically around the localities we had researched in depth in preceding years (including those communities themselves). We decided against a representative sample of the entire Kunene Region due to the size, poor road infrastructure, logistical constraints, and the lack of a list of communities that could serve as a sampling frame. Our approach allowed us to make use of the fact that fieldworkers were already known and trusted in the target areas. During the community visits, we elicited rules of water management and the composition of community-based organizational structures for water governance. Focus group discussions took place in public and included both female and male informants, of whom some were active in the committee responsible for community-based water resource management (Schnegg 2016b; Schnegg et al. 2016).

## Results

### Social networks and institutional multiplexity

My brief ethnographic description has already revealed that the communities are small, economically heterogeneous, and that people share more than water: they are kith and kin, share land for grazing as common property, help each other in everyday life, and belong to the same church. In short, they are linked in multiple different ways. In my theoretical discussion, I put emphasis on the overlap of different social fields, and particularly, the way in which economy and society are intertwined. As we have seen, institutional multiplexity measures the number of different contexts encompassed in a relationship between two individuals (Haythornthwaite 2001; Schweizer et al. 1996; Verbrugge 1979). If two actors interact in only one way, for instance by helping each other with herding, their social circles have little overlap. In this case, the institutional multiplexity of the relationships is one. If, however, both are also kin and borrow money from each other, the density of their ties is stronger and their social circles overlap to a larger degree. Again, we can count the number of different bases for their interactions, and their multiplexity is defined to be three.

Table 2 shows the multiplexity of ties across the seven communities in which we did ethnographic fieldwork. Any of the 776 relationships observed could entail a maximum of nine different transactions and a minimum of one. As we see in Table 2, almost 90% of the relationships contain more than one transaction and are thus multiplex. More than 50% of the relationships contain two or more transactions, indicating a high degree of network multiplexity (Schnegg and Linke 2015). This constitutes a multiplex social structure, and it is highly unlikely that interactions among water users only take into account rules and sanctions specifically regulating this resource. In contrast, (1) norms and codes of behavior that structure different social relations are likely to be blurred, and (2) further resource flows that take place within the multiplex social settings are likely to impact conduct with regard to water (Cleaver 2012). It is within these multiplex networks and multiple resource dependencies that people act economically when CBNRM is implemented.

**Table 2:** Institutional multiplexity of all network relationships (*N* = 776)

Institutional multiplexity	Number of relationships (*N*)	Percent of relationships (%)
Level 1	61	7.9
Level 2	324	41.8
Level 3	169	21.8
Level 4	89	11.5
Level 5	63	8.1
Level 6	50	6.4
Level 7	14	1.8
Level 8	5	0.6
Level 9	1	0.1
Sum	776	100

### Fixed boundaries

In her analysis, Ostrom has identified eight principles under which communities were found to manage communal resources successfully over long periods of time. The first principle reads “Clearly defined boundaries: the identity of the group and the boundaries of the shared resource are clearly delineated” (Cox et al. 2010; Ostrom 1990: 90).

In Namibia, after Independence, the design of new institutions was orchestrated from above, e.g., by the state and NGOs. During this phase, both organizational and institutional arrangements were negotiated, most importantly, by the Water Point Association (WPA) that includes all adult individuals who live in a place and want to use the public water point (Schnegg 2016b; Schnegg and Linke 2015). The Water Point Association puts the ideal of discrete user groups into practice. Membership in the WPA confers usage rights. At the same time, this does not imply that outsiders have no access at all. They may apply for temporary use rights. The differentiation between inside and outside is crucial and becomes most evident when examining the contributions to be made for the maintenance of the water point.

In Kunene, the rules of contribution were coded from 20 management plans. Management plans were formulated in community meetings organized and facilitated either by a contracted NGO or by extension officers from the Ministry of Agriculture, Water and Forestry (MAWF). In those documents, all communities agreed that contributions for insiders should be different from the contributions of outsiders. In all cases, outsiders are supposed to pay more, if their animals come to drink at the water point. The rate is usually more than double the insider fees (Schnegg and Bollig 2016). While people often asserted that they think it is rational to charge outsiders more money than insiders, we observed that in practice committees rarely or never insisted on such payments and/or found it difficult to procure them when they asked. There is a straightforward explanation.

In most communities, cattle are not herded during the day and move on their own. They habitually return to “their” water point in the evening or every second day to drink. However, if grazing is poor around the homestead, the cattle may keep on moving in search of better pastures. Where they find grazing, they next search for water. Although formal rules stipulate that a scarce good has to be used economically and that disincentives have to be put forward to deter potential free riders, these rules were rarely exercised. The informal acceptance of one’s neighbors’ cattle and the willingness to pay for them is motivated by other considerations; in the long run, everybody’s cattle will stray at times, and particularly so during the regular droughts. It is of the utmost advantage to know that almost nowhere will cattle that arrive at the well be turned away. This is particularly beneficial for wealthy livestock owners. Their abundant herds are most likely to be straying while cattle herds mainly consisting of cows are supervised more closely by herders. When animals do stray, a warning is sent to the livestock owner, and if this does not help, then the denial of water to cattle is a last option exercised in extremely few cases.

Densely woven networks of kinship and relatedness that include outsiders are the underlying reason for the reluctance to enforce extra levies on temporary well use by outsiders. In situations, where decisions on inclusion and exclusion have to be made, actors have to carefully weigh the costs of sanctioning against the value of actual exchange partners and the costs of increased and deregulated use of their well. In a context of multiple resource dependencies, this creates a sense of belonging and shared dependency that forms a social group on a larger scale than the immediate village. They foster and reinforce a pro-social behavior that is oriented toward long-term reciprocity instead of one-time monetary exchange. In short, multiplex social ties hinder the implementation of the original CBNRM principle of “fixed boundaries.” At the same time, they open other ways to cooperate in an extremely insecure environment.

### Cost sharing

Sharing the costs of water is one of the most salient problems in water governance. Since most pumps operate with diesel, the price of water is largely determined by the amount and price of diesel required for pumping it. Therefore, appropriators have to agree on a cost-sharing principle. The issue is central in Ostrom’s analysis (principle two), and she concluded that institutions are perceived to be fair if there is a “congruence between appropriation and provision rules” (Ostrom 1990: 90), and that institutions perceived to be fair are more likely to be successful. Her observation has been confirmed in a number of case studies (Klooster 2000; Pomeroy et al. 2001; Trawick 2001).

The handbooks of the Directorate of Rural Water Supply that were used to guide the process of crafting and designing institutions recommend “a rate per head of large or small stock, each member paying a certain rate per head of large or small [stock] accordingly, as to raise enough money to sustain the water point” (Namibia 2006: 8). We refer to this arrangement, which is based on a scientific approach, as the per head of cattle rule (or, alternatively, proportional equality).

In the communities we studied, two types of rules were applied. Among the 56 water management groups for which we have information, 25 (44.6%) agreed that individuals paid fees according to the number of livestock they owned (e.g., 2 N$ per head of cattle and 1 N$ per goat/sheep per month).^4^ Thus, the more water one uses, the more one pays. This fits the notion of proportional equality. In addition, seven communities (12.5%) used an attenuated form in which the rich paid more, but not exactly in proportion to the number of their livestock.^5^ However, in 24 communities (42.9%), we found an institutional regime in which all households paid the same (e.g., 100 N$ per household per month), and which was therefore called a flat-rate rule (or, alternatively, numerical equality).

Thus, only about half of the observations confirm the existing literature that a per head of cattle rule, which is in line with the CBNRM principles, should be instituted and is likely to be maintained (Cox et al. 2010; Ostrom 1990). To account for these results, we need to take the social structure and institutional multiplexity into account:

Wealth and bargaining power: Not surprisingly, wealthy herd owners opt for numerical equality. In contrast, poor households argue in the abstract that those who use more should also pay more. Across all communities, wealth differences result in patron–client relationships between the rich and the poor. In these relationships, those who own more use their bargaining power to push for an institutional regime favorable to them (i.e., a flat-rate rule) (Menestrey Schwieger 2015). Often, this is justified by pointing out that the higher burden on the poor is balanced out through other exchanges (e.g., milk, transportation) in different situations. The nature of social ties is key to understanding why they often succeed (Schnegg 2016b; Schnegg et al. 2016; Schnegg and Linke 2015).

Multiplexity of ties and norms of sharing: The communities each consist of fewer than 20 households, and people interact in multiple ways and roles. Thus, sharing water can hardly be separated from the remaining social and economic aspects of life. Sharing norms fosters a common belief that every household of the community needs to contribute to sustain collective goods and show cooperative commitment in multiple resource contexts (e.g., food, labor, pastures) (Schnegg 2015, 2016a). Given this interconnectedness in multiple networks, it is practically impossible for the less wealthy to force those who are better off to pay more than the rest if the latter refuse (Schnegg and Linke 2015).

Given both social dynamics, we would expect all communities to end up with a flat-rate rule. To understand why this is not the case, we have to take the state and its role into account. As we have seen, the state has an explicit preference for proportional equality (i.e., per head of cattle rule). Taking these dynamics together allows us to formulate a hypothesis: communities will only apply proportional equality when the state actively supports the poor and their interest in a proportional rule. In all other cases, the social dynamics described above, favor numerical equality. As the correlation between the two variables—state interventions, and the existence of proportional equality—reveals, the involvement of the state can explain the institutional outcome to a significant degree (Schnegg 2016b; Schnegg et al. 2016). In contrast, in communities where the state is only weakly involved the first two dynamics analyzed above are dominant and numerical equality prevails.

In sum, in a context of multiple resource dependencies, the existence and multiplexity of social ties hinder the application of Ostrom’s second principle, and the idea that water is an isolated economic good. Only when the state is active can it prevent communities slipping into a flat-rate rule.

### Formal sanctioning

In CPR theory, formal sanctions play a salient role and are seen as essential preconditions for successful resource management (Cox et al. 2010; Ostrom 1990: 90). In Ostrom’s work, sanctions are addressed by “principle 4.” Not surprisingly, the idea is taken up in the Namibian handbooks of the Directorate of Rural Water Supply which suggests, that “when rules for the use of the water point are decided upon (…), the WPA members should also decide on fines in cases where these rules are violated” (Namibia 2006: 48).

Our comparative documentary analysis from 21 communities reveals that all of them have specific, formal sanctions at their disposal. The regulations and sanctions mentioned in the management plan can roughly be categorized into two subject areas: first, in case community members do not pay their contributions; and second, in case the water point is not handled properly. In more than 60% of cases, violations of handling rules are sanctioned with a fine. Those fines range from 10 NAD for leaving the gates open up to 100 NAD^6^ for bathing at the water point, and can sometimes be different for community members and outside users. If community members refuse to pay contributions—an issue causing conflict in all of the communities we studied—graduated sanctions are listed in all documents. Some communities state explicitly that they would enforce exclusion with the help of the police (Schnegg and Linke 2015).

However, although the management plans provide detailed guidelines for how to enforce the rules, we almost never observed the application of sanctions in day-to-day water management. Although rules are broken now and then, during the entire fieldwork in seven communities we witnessed only a single case in which a fine was paid for breaking a “handling rule”. Regarding contributions, bending the rules—for example, failing to pay on time—is in fact a common practice. Violating the rules was seldom punished and in no case was a user excluded from access to the water point. To understand why, we need to take into account once more that people share multiple resources in multiplex social networks. When I asked Peter whether the community would punish someone for violating a rule, he replied:

Peter: There is just talk, but there is nothing going to happen. On paper, yes! But actually, it does not happen.

Michael: Any reason?

Peter: Tomorrow, my cattle will also come there. If there is no grazing here, my cattle will be there. And then, they will treat me the same way. So, that is one thing why we are careful to punish.

Michael: So it is better to ...

Peter: ... be friendly with the people.

Peter: And, there is another thing, too. You know, we grew up together. We have a long history of living in this place. It is difficult to punish those who are close to you. Like me, I cannot punish James for breaking the fences. His father is my uncle.^7^

As we have seen, community members not only share water but also food, livestock, ancestries, and other goods and experiences. In the conversation between Peter—a well-off pastoralist in his 40s—and me, he explains the inability to punish with two arguments. First, people interact in long-term relationships and it is likely that their respective roles may change soon: those who suffer from a rule violation may soon be violators themselves. In this context, Peter does not want to get into conflict over a relatively minor issue, and argues that it is better to do nothing than to try to apply formal sanctions. Second, he explains that people share manifold experiences through their living in the same area for long time. This makes it difficult to punish someone to whom one is close. The situation gets even more difficult when kinship is involved, because the role relationship may make any punishment unappropriated. In the case he refers to, his nephew had ruined the fence of the communal water point with a donkey cart, but Peter was hesitant to go to his uncle (whom he is expected to respect) to make a formal accusation.

This multiplexity of sharing restricts the agency of actors who cannot separate sharing water from other past or synchronous interactions. Thus, it is hard, and often impossible, to apply a formal sanction to someone who is at the same time one’s respected kinsman, a neighbor, and the person with whom one shares food regularly (Schnegg and Linke 2015). The normative expectations of subordination and respect entailed in some kinship ties restrict the behavior of people to a large degree and “overrule” the water-sharing relation in many regards. Thus, even if they would want to, people could not act as if they “only” share water.

However, multiplexity offers distinctive opportunities for monitoring social behavior and controlling resources. Dense and multiplex relations allow a rapid spread of information and provide detailed knowledge about the social and economic situations of community members. This allows others to contextualize their behavior and their breaking or bending of the rules. Communication and information become means of social control (Schnegg and Linke 2015). As Olga, who serves as a secretary in a community around Fransfontein, explained to me:

“Some houses here are very poor. They only have five goats. Often, they cannot pay the water fees. How could we punish them? There is nothing to take. But you know, if I see that their daughter has sent money from Windhoek and they buy nice food, like macaroni and soup [MS: a highly sought-after and expensive dish], I go visit them. I will ask at last some part of the contribution. And at that time, they are going to give.”^8^

As we see, it is generally accepted that some households find it difficult to pay their share. However, the visibility of resources is high in a face-to-face community. Therefore, the costs for not contributing would be very high in a situation where others know that financial resources are at hand. As people put it, “If you have, you must give” (Schnegg 2015, 2016a). When having, for most households (and especially the poor), the threat of isolation from a larger network or community sphere is more significant than any gain that might result from the violation of a rule.

Taken together, we see that people cannot separate the sharing of water from the sharing of ancestries, food, and work. This discourages the application of formal sanctions while opening other means of maintaining institutional regimes.

## Discussion and conclusion

During recent decades, the ways natural resources are managed in the global South have changed significantly. While the state withdrew from managing resources actively, it framed the ways communities would do so. One of the dominant frameworks, CBNRM, was informed by common-pool theory, and emphasized principles such as fixed boundaries, proportional cost sharing, and formal sanctioning (Saunders 2014). In common-pool theory, those principles have been recognized as salient factors for successful, sustainable, and just natural resource management. Common-pool theory itself largely applies a rational actor model and typically assumes that those actors interact with a single issue in mind. In Namibia, CBNRM was implemented using blueprints which were locally adopted and designed (Bollig and Menestrey Schwieger 2014; Jones 2010; Jones and Murphree 2001; Schnegg 2016b; Schnegg and Linke 2016).

The evidence presented here first of all shows that three salient CBNRM principles—fixing boundaries, sharing costs proportional to use, and formal sanctioning—are rarely put into practice: boundaries are not fixed, costs are not shared proportional to use, and formal sanctions are almost never applied. As I have shown, the nature of social networks can help to explain why. While institutional embeddedness was recognized in economic anthropology some time ago, its importance for natural resource management has long been overlooked in the dominant economic debates. Only recently has critical institutionalism emphasized the relationships between institutions and social ties (Benjaminsen and Lund 2002; Cleaver 2012; Cleaver and de Koning 2015).

However, until now, we have lacked a sound conceptual framework and a methodological technique for studying the relationship of society and resource governance. To overcome this gap and to access “embeddedness” more systematically than before, I have proposed and further developed the notion of institutional multiplexity. Institutional multiplexity describes the degree to which the natural resource economies are embedded in multiple social fields. The concept derives from on network theory and offers, for the first time, both a theoretical guide and a measurement tool to access and compare social embeddedness systematically.

The analysis of water governance in seven Namibian communities reveals that institutional multiplexity is a salient feature of their social structure. As we have seen, people depend on sharing multiple resources. They not only share water but also interact in up to eight other domains. Each of these domains is governed by its own norms, rules, and expectations (Schnegg 2015, 2016a). Thus, a salient implicit assumption of the CBNRM model—that people live institutions and take decisions over one isolated fields—is not met.

As I have shown, institutional multiplexity has far reaching consequences for individuals’ conduct. As the ethnographic analysis reveals, people find it impossible to treat water as a separate social field; other roles circumscribe how people interact with one another in this area, as well as others. For example, while kinship and other network ties lend power to some to leverage specific cost-sharing regimes, they restrict the agency of others to apply formal institutions. After all, society is constituted in significant part by economic interactions among its members. In all three cases discussed—fixing boundaries, sharing costs proportional to use, and formal sanctioning—institutional multiplexity can explain, to a significant degree, why institutions are practiced other than designed.

Whether and when social networks provide means to foster cooperation to govern resources successfully is still a young debate. Focusing on social capital, Bodin and Crona (2008) have shown that high network density and connectivity does not always lead to sustainable resource management (Bodin and Crona 2008). In a similar vein, Crona et al. have found that leadership may be a more valid explanation of success than social capital (Crona et al. 2017). In contrast, Barnes et al. (2016) revealed that social networks (mostly in terms of their homophily) are positively related to the diffusion of sustainable behaviors and environmental outcomes. In sum, while the importance of the relationship between network properties and environmental outcomes is increasingly recognized, the precise directions and effects involved remain to be explored (Bodin 2017; Henry and Vollan 2014).

My findings about institutional multiplexity and resource management have political implications as well. They call for caution in developing blueprints from Ostrom’s work (Ostrom 2009; Saunders 2014). Fixing boundaries, proportional cost sharing, and formal sanctions, which are a salient part of almost all CBNRM programs, are unlikely to be effective and practically applied in social environments characterized by manifold resource dependencies and institutionally multiplex networks and ties. Here, institutional approaches that single out domains (such as water) without recognizing interlinkages with other social fields are prone to fail. At the same time, we have to recognize that institutional multiplexity provides alternative principles of social control which can effectively support collective resource management. In all the cases I describe, people have access to water and regulate it successfully in myriad, and often ad hoc ways. Further developments of CBNRM should take the flexibility which networks provide into account.

For development practices this might advise to explore, how formal institutional blueprints are likely to be applied in a specific locale. Our research suggests that in situations in which resource dependency and institutional multiplexity are high and communities are small and based on face-to-face interactions, informal and ad hoc institutions emerge. Under those circumstances, it might be useful to explore the distributive consequences these emerging and culturally embedded institutions have. If they disadvantage social groups, it might be more advisable to support those groups directly (e.g., through monetary subsidiaries) than trying to push through formal institutional solutions that are favored because they are seen as democratic and fair.

## Footnotes

1 The tremendous importance of social networks is increasingly recognized by mainstream institutionalism as well (Bodin 2017; Bodin and Crona 2008; Crona et al. 2017; Henry and Vollan 2014; Janssen et al. 2006).

2 In contrast, Ostrom defined institutional diversity as how distinct institutions regulate different social situations, independently of whether they overlap or not (Ostrom 2005). In her analysis she offers a very elaborate framework for exploring and explaining the similarities and differences between institutional solutions.

3 Today, experimental economics question the usefulness of continuing the conceptualization of decision-making as largely rational and egoistic. At the same time, most experiments still test decisions in isolated and anonymous interactions (Fehr et al. 2002; Henrich 2004; Ostrom and Walker 2003).

4 Four of the 60 communities in the sample could not be included because some relevant data are incomplete or missing.

5 The following analysis treats the six cases with an attenuated proportional rule as cases of a proportional rule.

6 At the time of the fieldwork 100 NAD exchanged for approximately 7 Euro.

7 Interview conducted in the Fransfontein area, March 2014.

8 Interview conducted in the Fransfontein area, March 2014.
